# Clinicopathologic Features and Treatment Outcomes in Patients with Stage I, High-Risk Histology or High-Grade Endometrial Cancer after Primary Staging Surgery: A Taiwanese Gynecologic Oncology Group Study

**DOI:** 10.3390/jcm7090254

**Published:** 2018-09-04

**Authors:** Ming-Shyen Yen, Tze-Ho Chen, Yu-Min Ke, Keng-Fu Hsu, Jen-Ruei Chen, Mu-Hsien Yu, Hung-Chun Fu, Chia-Yen Huang, An-Jen Chiang, Chao-Yu Chen, Sheng-Mou Hsiao, Yuen-Yee Kan, Fu-Shing Liu

**Affiliations:** 1 Department of Obstetrics and Gynecology, Taipei Veterans General Hospital, Taipei 112, Taiwan; msyen@vghtpe.gov.tw; 2 Department of Obstetrics and Gynecology, Changhua Christian Hospital, Changhua 500, Taiwan; tzeho@cch.org.tw; 3 Department of Obstetrics and Gynecology, Taichung Veterans General Hospital, Taichung 407, Taiwan; yuminke@hotmail.com; 4 Department of Obstetrics and Gynecology, National Cheng Kung University Hospital, Tainan 704, Taiwan; d5580@mail.ncku.edu.tw; 5 Department of Obstetrics and Gynecology, Mackay Memorial Hospital, Taipei 104, Taiwan; cremaster4471@gmail.com; 6 Department of Obstetrics and Gynecology, Tri Service General Hospital, Taipei 114, Taiwan; hsienhui@ms15.hinet.net; 7 Department of Obstetrics and Gynecology, Kaohsiung Chang Gung Memorial Hospital, Kaohsiung 833, Taiwan; allen133@adm.cgmh.org.tw; 8 Department of Obstetrics and Gynecology, Cathay General Hospital, Taipei 106, Taiwan; bagiao2003@gmail.com; 9 Department of Obstetrics and Gynecology, Kaohsiung Veterans General Hospital, Kaohsiung 813, Taiwan; ajchiang490111@gmail.com; 10 Department of Obstetrics and Gynecology, ChiaYi Chang Gung Memorial Hospital, ChiaYi 613, Taiwan; chauyuchen@gmail.com; 11 Department of Obstetrics and Gynecology, Far Eastern Memorial Hospital, New Taipei City 220, Taiwan; smhsiao2@gmail.com; 12 Department of Obstetrics and Gynecology, Yuan’s General Hospital, Kaohsiung 802, Taiwan; kyy_gyn@yahoo.com.tw; 13 Department of Obstetrics and Gynecology, Show Chwan Memorial Hospital, Changhua 500, Taiwan

**Keywords:** clinicopathologic features, outcomes, stage I, high-risk histology, high-grade, endometrial cancer

## Abstract

To investigate the clinicopathological features and treatment outcomes in patients with stage I, high-risk endometrial cancer. Patients with International Federation of Gynecology and Obstetrics stage I, papillary serous, clear cell, or grade 3 endometrioid carcinoma treated between 2000 and 2012 were analyzed for the clinical and pathological factors in relation to prognosis. A total of 267 patients (stage IA; *n* = 175, stage IB; *n* = 92) were included. Among the clinicopathological features, stage and age were significant prognostic factors. The recurrence rate and overall survival for stage IB versus IA were 22.8% versus 9.1% (*p* = 0.003) and 149.7 months versus 201.8 months (*p* < 0.001), respectively. The patients >60 years of age also had a higher recurrence rate (21.7% versus 9.7%, *p* = 0.008) and poorer survival (102.0 months versus 196.8 months, *p* = 0.001) than those ≤60 years of age. Distant recurrence (64.9%) occurred more frequently than local recurrence (24.3%) and local combined with distant recurrence (10.8%) (*p* < 0.001). The postoperative treatment modality had no impact on tumor recurrence rate, recurrence site, or overall survival. Distant recurrence is a major cause of treatment failure in patients with stage I, high-risk endometrial cancer. However, current adjuvant treatment appeared to have little effect in preventing its occurrence.

## 1. Introduction

Most early stage endometrial cancers are curable with surgical resection alone and a favorable outcome can be expected. However, disease recurrence may occur and affect patient outcomes when clinical or pathological adverse factors are present. The conventional risk factors for recurrence in early stage endometrial cancer include tumor stage, increasing patient age, tumor size, high-risk histological type, grade 3 adenocarcinoma, myometrial invasion and lymphovascular space invasion (LVSI) [1,2,3,4], all of which have been associated with a higher risk of recurrence and nodal metastasis [2,4,5,6,7]. Among these adverse factors, a Gynecologic Oncology Group study reported that tumor grading was the greatest determinant of recurrence, with a relative risk of 15 for grade 3 adenocarcinoma [8]. In addition, an analysis of 104 patients with 1988 International Federation of Gynecology and Obstetrics (FIGO) stage IC, grade 3 endometrial cancer who were registered but excluded from the Postoperative Radiation Therapy in Endometrial Carcinoma (PORTEC) trial, also demonstrated that grade 3 differentiation was the most important adverse prognostic factor for relapse and death [9]. Furthermore, a recent study of 521 patients with stage I endometrioid endometrial carcinoma reported that histologic grade was the only risk factor associated with tumor recurrence in patients with stage IB. The 5-year recurrence-free survival rates in this group of patients were 94% for grade 1, 79% for grade 2 and 74% for grade 3 [10].

Despite being the most important risk factor for tumor recurrence, the current treatment strategies for patients with stage I, grade 3 endometrial cancer after surgery are varied and inconclusive. The postoperative adjuvant treatments can range from observation only, radiation with external beam radiotherapy (EBRT) or vaginal brachytherapy (VBT), to radiation plus systemic chemotherapy (CT) [11]. The use of different criteria of the risk factors to determine the postoperative treatment strategy may be the reason for the varying clinical practices [12,13]. In addition, most of the clinical trials on early-stage, high-risk disease have included patients with stage I and IIA [2,6,14]. Combining different cancer stages may interfere with the accuracy of the analysis and lead to different conclusions.

In this study, we focused on patients with high-risk stage I endometrial cancer, including the high-risk histological type or grade 3 endometrioid adenocarcinoma. The aim of this study was to investigate whether different postoperative treatment modalities affect patient outcomes. Other clinicopathologic risk factors were also analyzed to evaluate their prognostic significance in this group of patients.

## 2. Materials and Methods

### 2.1. Patients and Study Design

This was a multicenter, retrospective study approved by the Institutional Review Boards of each participating center. Patients with 2009 FIGO stage I, papillary serous, clear cell, or grade 3 endometrial carcinoma treated between January 2000 and December 2012 were enrolled for analysis. Those who were staged as IA and IB before the 2009 FIGO staging system were defined as having stage IA and those with old stage IC were classified as having stage IB. All patients received an open or laparoscopic staging operation as the primary treatment, which included total abdominal hysterectomy (TAH) or laparoscopic-assisted vaginal hysterectomy (LAVH) plus bilateral pelvic lymph node dissection (BPLND) with or without para-aortic lymph node sampling or dissection (PALNS/D). Age, surgical type, pathological features, postoperative treatment modality, recurrence site and overall survival time were recorded according to the chart records and the follow-up system. Patients with other concomitant malignancies or uterine carcinosarcoma were excluded. The study was conducted in accordance with the Declaration of Helsinki and the protocol was approved by the Institutional Review Board of each participating hospitals.

To analyze the significance of the clinical and pathological factors on patient outcomes, we classified the surgical types into two groups irrespective of whether bilateral salpingo-oophorectomy (BSO) was performed during surgery (i.e., TAH or LAVH + BPLND and TAH or LAVH + BPLND + PALNS/D). The patients were stratified by age into two groups: those ≤60 years and those >60 years of age. Tumor size was classified into those <2 cm and those ≥2 cm in diameter. According to the presence of LVSI, the patients were divided into negative and positive groups. For postoperative treatment, the patients were divided into four groups: observation only, radiotherapy (RT) alone with either EBRT or VBT or both, chemotherapy alone and chemotherapy plus radiotherapy.

Tumor recurrence was diagnosed based on pathological verification or image studies including computerized tomography, positron emission tomography, magnetic resonance imaging and whole-body bone scan. Based on the site of recurrence, the patients were divided into three groups: local recurrence (pelvis or vagina), distant recurrence (recurrent site beyond the pelvis) and local plus distant recurrence. Overall survival was calculated from the date of primary surgery to December 2016 or the date of death.

### 2.2. Statistical Analysis

Associations between stage, age, primary surgical type, tumor size and presence of LVSI and recurrence were analyzed using univariate and multivariate logistic regression analysis. The influence of the various clinicopathological features on overall survival was evaluated using the Kaplan-Meier method with the log-rank test. Fisher’s exact test was used to analyze the relationship between postoperative management and the site of recurrence. Recurrence site patterns were compared using the Z-test. A *p*-value of < 0.05 was considered to be statistically significant. IBM SPSS Statistics for Windows, version 24.0 (IBM Corp., Armonk, NY, USA) was used for the statistical analyses.

## 3. Results

A total of 267 patients were eligible for the analysis. Their clinicopathologic features are presented in Table 1. Overall, 175 patients were at stage IA and 92 patients were at stage IB. In addition, 203 patients had grade 3 endometrioid carcinoma, 37 patients had papillary serous carcinoma and 27 patients had clear cell carcinoma. The median age at diagnosis was 57 years (range: 31–83 years). The median follow-up period was 67.9 months (range: 4–201 months). Recurrence occurred in 37 patients, with a recurrence rate of 13.9%.

**Figure 1:**
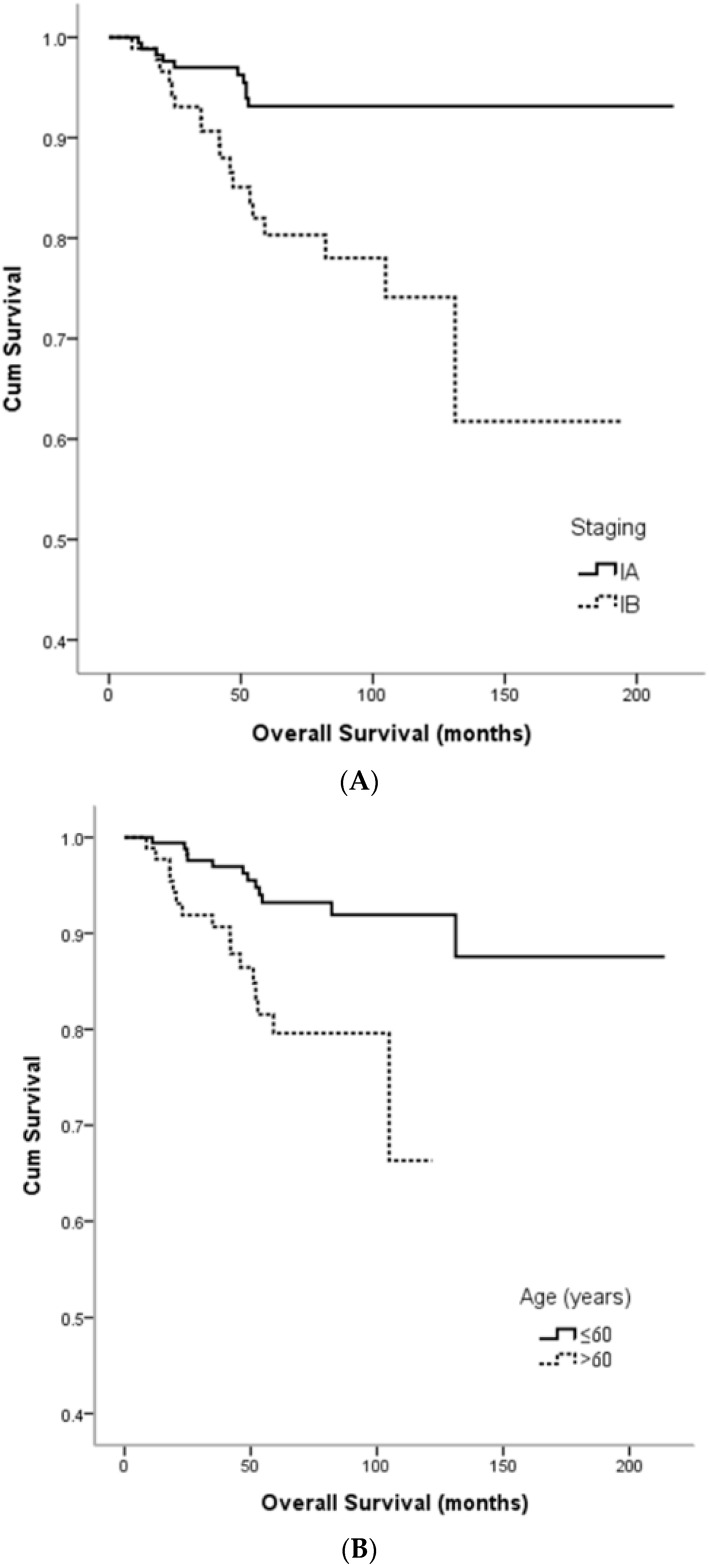
Kaplan-Meier survival estimates according to stage and age. (**A**) Overall survival according to stage (log-rank: IA vs. IB, *p* < 0.001); (**B**) Overall survival according to age (log-rank: ≤60 years vs. >60 years, *p* = 0.001).

Among the clinicopathologic features, stage and age were significant prognostic factors for tumor recurrence in both univariate and multivariate analyses (Table 2). Patients with stage IB disease were associated with a higher recurrence rate than those with stage IA disease (22.8% vs. 9.1%, *p* = 0.003, in both univariate and multivariate analyses) and also a worse overall survival (149.7 months vs. 201.8 months, *p* < 0.001) (Figure 1A). Patients >60 years of age had a higher recurrence rate than those who were ≤60 years of age (21.7% vs. 9.7%, *p* = 0.008 in univariate analysis and *p* = 0.022 in multivariate analysis) (Table 2). Similarly, they also had worse overall survival than the younger patients (102.0 months vs. 196.8 months, *p* = 0.001) (Figure 1B). Tumor size (<2 cm vs. ≥2 cm) and the status of LVSI (negative vs. positive) were not significantly associated with tumor recurrence or overall survival. 

**Figure 2:**
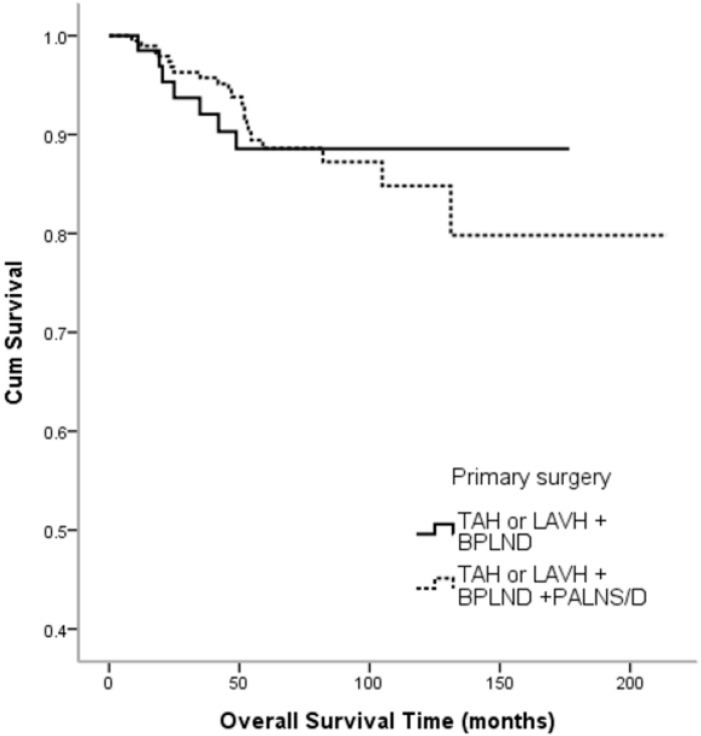
Kaplan-Meier survival estimates according to primary surgical types (log-rank: TAH or LAVH + BPLND vs. TAH or LAVH + BPLND + PALNS/D, *p* = 0.884). TAH: total abdominal hysterectomy; LAVH: laparoscopic-assisted vaginal hysterectomy; BPLND: bilateral pelvic lymph node dissection, PALNS/D: para-aortic lymph node sampling or dissection.

Seventy patients received TAH or LAVH + BPLND and 197 patients received TAH or LAVH + BPLND + PALNS/D. BSO was performed in 263 patients. There were no significant differences in recurrence rate (*p* = 0.778) (Table 2) or overall survival time (*p* = 0.884) (Figure 2) between these two patient groups. 

Among the 37 patients with disease recurrence, nine (24.3%) had local recurrence, 24 (64.9%) had distant recurrence and four (10.8%) had both local and distant recurrence. The frequency of distant recurrence was significantly higher than both local recurrence (*p* < 0.001) and local plus distant recurrence (*Z*-test, *p* < 0.001). 

**Figure 3:**
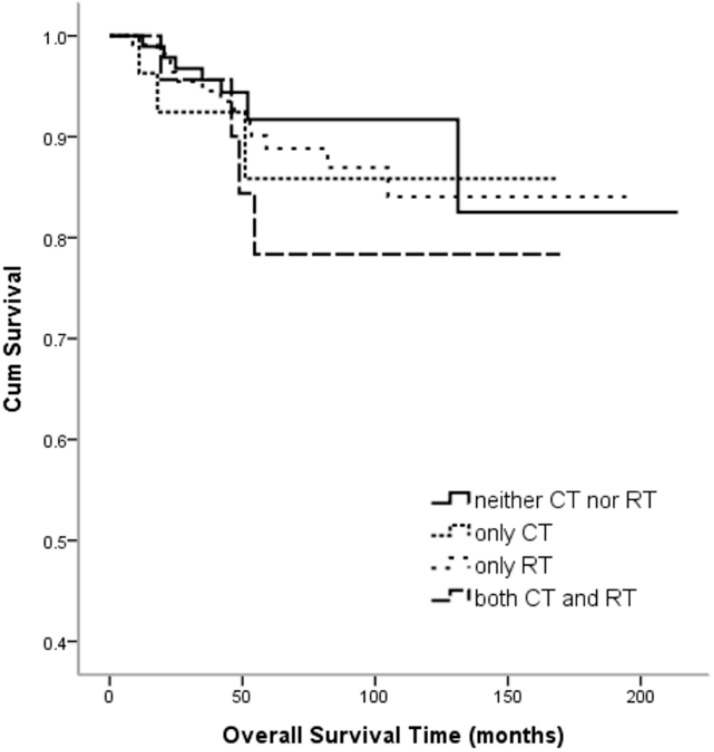
Kaplan-Meier survival estimates according to postoperative adjuvant therapies (log-rank, *p* = 0.621). CT: chemotherapy; RT: radiotherapy.

After the primary staging surgery, 102 patients (38.2%) were under observation with no further adjuvant treatment, 114 patients (42.7%) received radiotherapy and 28 patients (10.5%) received chemotherapy as postoperative adjuvant treatment. Chemotherapy plus radiotherapy was given to 23 patients (8.6%). No difference was noted in the recurrence rate (*p* = 0.440) (Table 3) and recurrence site (*p* = 0.390) (Table 4) among the four treatment modalities. There was also no difference in overall survival (*p* = 0.621) (Figure 3) among the four patient groups.

## 4. Discussion

Patients with early-stage, high-risk histology, or grade 3 endometrioid endometrial cancer are known to have a higher recurrence rate and worse survival. Clinically these patients are classified into either high-intermediate risk or high-risk groups according to whether other risk factors are present [15]. Given the poor outcomes, postoperative adjuvant treatment is recommended for this group of patients. In the PORTEC-1 clinical trial, EBRT was confirmed to be effective in reducing the locoregional recurrence rate in stage I patients with intermediate or high-intermediate risk [1]. The PORTEC-2 study also observed this effect of VBT for patients with high-intermediate risk [6]. However, both studies concluded that neither EBRT nor VBT had impact on overall survival compared with the patients who did not receive adjuvant therapy. A long-term follow-up study of the PORTEC-1 trial further confirmed that EBRT had no benefit in patients with high-intermediate risk on 15-year overall survival and endometrial cancer-related death [16]. 

Since both the PORTEC-1 and PORTEC-2 trials excluded patients with FIGO 1988 stage IC and grade 3 disease (i.e., FIGO 2009 stage IB, grade 3), the adjuvant treatment strategy in this specific group of patients has yet to be determined.

In this retrospective study, we focused on patients with stage I endometrial cancer with high-risk histology or grade 3 adenocarcinoma. Among the four analyzed clinicopathological features, only myometrial invasion (stage IB) and age (>60 years) exhibited prognostic significance. The other two conventional risk factors, tumor size and LVSI, had no longer prognostic role in this group of patients.

Distant metastasis is regarded to be the main cause of treatment failure in patients with early stage, high-risk histology or grade 3 endometrial cancer even though who have received postoperative radiation therapy [17]. In the current study, we also observed that distant metastasis occurred in about 75% of the patients with recurrence. In addition, postoperative chemotherapy or chemotherapy plus radiotherapy did not affect the recurrence rate or overall survival, although the percentage of these patients in this study was relatively low (19%).

Given that distant recurrence is a major obstacle in improving the outcomes of patients with early-stage, high-risk endometrial cancer, postoperative adjuvant chemotherapy has been recommended to prevent its occurrence [18,19]. However, the effect of chemotherapy on preventing distant recurrence has shown inconsistent results in several relevant studies. A large retrospective study that analyzed 11,746 stage IB and II endometrial cancer patients with either papillary serous, clear cell, or grade 3 adenocarcinoma, concluded that the addition of adjuvant chemotherapy to radiation was associated with improved overall survival [20]. However, the Gynecology Oncology Group Study (GOG249) conducted a randomized phase 3 trial of pelvic radiation therapy versus vaginal cuff brachytherapy followed by paclitaxel plus carboplatin chemotherapy in patients with high-risk, early-stage endometrial cancer. The initial report revealed that VBT plus CT was not superior to EBRT in terms of 36-month recurrence-free survival and overall survival. Moreover, no significant difference was observed between the two arms in vaginal or distant failure [21]. Recently, the PORTEC-3 study reported that adjuvant chemotherapy given during and after radiotherapy for high-risk endometrial cancer did not improve 5-year overall survival. The study also included patients with stage IB, grade 3 adenocarcinoma and stage I with serous or clear cell histology. Therefore, addition of chemotherapy to postoperative radiation was not recommended as a standard for patients with stage I-II, high-risk disease [22]. Finally, a systematic review and meta-analysis compared adjuvant chemoradiotherapy versus radiotherapy alone in stage I-III high-risk endometrial cancer. It also revealed no significant differences in overall survival, local recurrence rate, or distant metastasis rate although improvement of five-year progression free survival and five-year cancer-specific survival were observed [23]. Taken together, the optimum treatment strategy for patients with high-risk, early-stage endometrial cancer has yet to be elucidated. 

Endometrial cancer is known as a molecular heterogeneous disease [24]. Through a comprehensive integration of genomic characterization, The Cancer Genome Atlas (TCGA) has defined four distinct subgroups in this neoplasm: POLE ultramutated, microsatellite instability (MSI), hypermutated, copy-number low and copy-number high with frequent p53 mutations [25]. In an analysis of the PORTEC cohorts, integrating molecular and clinicopathological factors was shown to improve the prognostic assessment of patients with early-stage endometrial cancer. In this study, p53-mutant tumors were significantly associated with grade 3 differentiation and were independent prognostic factors for distant recurrence, overall and disease-specific survival. MSI and copy-number low tumors had intermediate prognosis. The POLE-mutant tumors had a favorable prognosis comparing to the other three subgroups [26]. Similar results were also observed in other studies [27,28].

The Gynecologic Cancer Steering Committee of the US National Cancer Institute recently integrated molecular and/or histologic stratification into endometrial cancer management [29]. Through molecular classification, several target genes and molecular pathways in this tumor were identified, including DNA repair, hormone-related pathways, ERBB2/HER2, PI3K/AKT/mTOR signaling, the WNT pathway, immune-related pathways and obesity-driven targets. In addition, it is found that endometrial cancer cells can make cell-to-cell communication via exosomes-transferred microRNAs to modify tumor biology [30]. A number of microRNAs are believed to involve tumor growth, proliferation, invasion, metastasis and chemoresistance [31]. These findings are expected to provide insight into clinical trials of appropriately designed therapeutic strategies in selected patients.

Another potential strategy in cancer treatment is targeting the genes involving in the tumor circadian clocks [32]. Alterations of the circadian genes have been observed in various kinds of cancer including endometrial cancer [33,34,35,36,37]. By manipulating the tumor circadian clocks the tumor growth can slow down and clinically it may help to establish more appropriate anticancer approaches [32].

In summary, patients with stage I, high-risk endometrial cancer have a tendency to suffer from distant metastasis. The effect of current adjuvant therapies on preventing its occurrence remains inconclusive and seems to be unsatisfactory. Identifying new therapeutic approaches based on molecular and clinicopathologic features to develop potential candidate antitumor agents in selected patients is of vital importance.

## Figures and Tables

**Table 1:** Clinicopathologic features of patients (*n* = 267).

Variables	*n*	(%)
FIGO Stage		
IA	175	(65.5)
IB	92	(34.5)
Median age (year) (range)	57 (31–83)	
≤60	175	(65.5)
>60	92	(34.5)
Median follow-up (m) (range)	67.9 (4–201)	
Primary surgery		
TAH (LAVH) + BPLND	70	(26.2)
TAH (LAVH) + BPLND + PALNS/D	197	(73.8)
BSO		
Yes	263	(98.5)
No	4	(1.5)
Histology		
Endometrioid	203	(76.0)
Papillary serous	37	(13.9)
Clear cell carcinoma	27	(10.1)
Tumor size		
<2 cm	80	(30.7)
≥2 cm	181	(69.3)
LVSI		
Positive	65	(24.6)
Negative	199	(75.4)
Postoperative treatment		
Observation	102	(38.2)
R/T	114	(42.7)
C/T	28	(10.5)
R/T + C/T	23	(8.6)
Recurrent site		
Local	9	(24.3)
Distant	24	(64.9)
Local + Distant	4	(10.8)

FIGO: International Federation of Gynecology and Obstetrics; TAH: total abdominal hysterectomy; LAVH: laparoscopic-assisted vaginal hysterectomy; BPLND: bilateral pelvic lymph node dissection, PALNS/D: para-aortic lymph node sampling or dissection; LVSI: lymphovascular space invasion; BSO: bilateral salpingo-oophorectomy.

**Table 2:** Univariate and multivariate logistic regression analyses of the prognostic factors for recurrence.

Prognostic Factor	Univariate Analysis			Multivariate Analysis		
	OR	95% CI	*p*	OR	95% CI	*p*
Stage (IB)	2.939	1.448–5.967	0.003	3.305	1.488–7.338	0.003
Tumor size (≥2 cm)	1.174	0.537–2.567	0.687	0.843	0.356–1.996	0.698
LVSI (+)	1.024	0.454–2.307	0.955	0.772	0.321–1.854	0.562
Age (>60)	2.582	1.277–5.220	0.008	2.342	1.128–4.863	0.022
Surgery with PALNS/D	1.123	0.502–2.514	0.778	1.190	0.510–2.778	0.688

Reference category: tumor size (<2 cm); LVSI (−); age (≤60); surgery (without PALNS/D). PALNS/D: para-aortic lymph node sampling or dissection; OR: odds ratio; CI: confidence interval; LVSI: lymphovascular space invasion; PALNS/D: para-aortic lymph node sampling or dissection.

**Table 3:** Postoperative treatments and the recurrence rate.

Postoperative Treatment	Recurrence					
	No	(%)	Yes	(%)	Total	(%)
Neither CT nor RT	91	89.2%	11	10.8%	102	100.0%
Only CT	23	82.1%	5	17.9%	28	100.0%
Only RT	98	86.0%	16	14.0%	114	100.0%
Both CT and RT	18	78.3%	5	21.7%	23	100.0%
Total	230	86.1%	37	13.9%	267	100.0%

Fisher’s Exact Test: *p* = 0.440; CT: chemotherapy; RT: radiotherapy.

**Table 4:** Postoperative treatments and the recurrent site.

Postoperative Treatment	Recurrence Site 1		Recurrence Site 2		Recurrence Site 3		Total	
	*n*	(%)	*n*	(%)	*n*	(%)	*n*	(%)
Neither CT nor RT	3	27.3%	5	45.5%	3	27.3%	11	100.0%
Only CT	0	0.0%	5	100.0%	0	0.0%	5	100.0%
Only RT	4	25.0%	11	68.8%	1	6.3%	16	100.0%
Both CT and RT	2	40.0%	3	60.0%	0	0.0%	5	100.0%
Total	9	24.3%	24	64.9%	4	10.8%	37	100.0%

Recurrence site 1: pelvic recurrence; Recurrence site 2: distant recurrence; Recurrence site 3: pelvic + distant recurrence Fisher’s Exact Test: *p* = 0.390, CT: chemotherapy; RT: radiotherapy.
